# Respirator Donning in Post-Hurricane New Orleans

**DOI:** 10.3201/eid1305.061490

**Published:** 2007-05

**Authors:** Kristin J. Cummings, Jean Cox-Ganser, Margaret A. Riggs, Nicole Edwards, Kathleen Kreiss

**Affiliations:** * National Institute for Occupational Safety and Health, Morgantown, West Virginia, USA; † Centers for Disease Control and Prevention Epidemic Intelligence Service, Atlanta, Georgia, USA; ‡ National Institute for Occupational Safety and Health, Cincinnati, Ohio, USA

**Keywords:** Respirators, pandemic, disease transmission, molds, natural disasters, Hurricane Katrina, New Orleans, research

## Abstract

We evaluated correctness of N95 filtering facepiece respirator donning by the public in post-hurricane New Orleans, where respirators were recommended for mold remediation. We randomly selected, interviewed, and observed 538 participants, using multiple logistic regression for analysis. Only 129 (24%) participants demonstrated proper donning. Errors included nose clip not tightened (71%) and straps incorrectly placed (52%); 22% put on the respirator upside down. Factors independently associated with proper donning were as follows: ever having used a mask or respirator (odds ratio [OR] 5.28; 95% confidence interval [CI], 1.79–22.64); ever having had a respirator fit test (OR 4.40; 95% CI, 2.52–7.81); being male (OR 2.44; 95% CI, 1.50–4.03); Caucasian race (OR 2.09; 95% CI, 1.32–3.33); having a certified respirator (OR 1.99, 95% CI 1.20–3.28); and having participated in mold clean-up (OR 1.82; 95% CI,1.00–3.41). Interventions to improve respirator donning should be considered in planning for influenza epidemics and disasters**.**

## Abstract 2

Most participants did not properly don an N95 FF respirator

Many respirators certified by the National Institute for Occupational Safety and Health (NIOSH), particularly disposable N95 filtering facepiece respirators (N95 FF respirators [Figure 1]), are available to the public. The certification indicates that the respirator material will perform at a given filter efficiency (*1*). Because proper fit is also necessary for respirator function, US regulations state that an employer who requires workers to wear respirators must establish a respiratory protection program that covers respirator selection and maintenance, fit testing, and worker instruction (*2*). Although nonoccupational respirator use has not been well studied, members of the public who use respirators may be less likely than workers in a respiratory protection program to achieve a proper fit, given lack of formal training (*3*).

**Figure 1:**
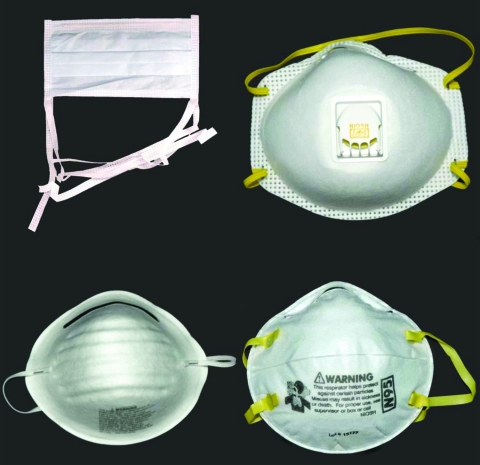
Noncertified masks and certified respirators. A surgical mask (upper left) and a dust mask (lower left) are examples of disposable masks that are not designed to filter small particles and that are not certified by the National Institute for Occupational Safety and Health (NIOSH). The disposable N95 filtering facepiece respirators pictured on the right (with exhalation valve, upper right; without exhalation valve, lower right) are made of material certified by NIOSH to filter 95% of 0.3-μm diameter particles and bear the NIOSH name and “N95” filter identification. The European FFP2 respirator is most analogous to the N95 filtering facepiece respirator. NIOSH also certifies more expensive reusable respirators (not pictured), which can be fitted with disposable cartridges that filter particles. Reusable respirators may cover the face from the bridge of the nose to the chin (half-face) or from the forehead to the chin (full-face).

Public health agencies have recommended N95 FF respirators to members of the public for some situations. Such occasions have included after major floods, for potential heavy exposure to bioaerosols in water-damaged buildings (Grand Forks, North Dakota, 1997; eastern North Carolina after Hurricane Floyd, 1999) (*4*), and for settings that pose a risk for airborne transmission of infection, such as during the severe acute respiratory syndrome (SARS) epidemic (for select patients at risk of acquiring the infection and for persons visiting patients with SARS) (*5*–*7*). There is also a longstanding recommendation for N95 FF respirator use for visitors of hospitalized patients with tuberculosis (*8*). The US Department of Health and Human Services (HHS) currently recommends that persons living in or visiting an area affected by avian influenza A (H5N1) wear N95 FF respirators when in contact with birds in an enclosed environment (*9*).

In the fall of 2005, after the unprecedented flooding in New Orleans, Louisiana caused by Hurricanes Katrina and Rita, public health officials recommended that members of the public use N95 FF respirators when cleaning or remediating mold-contaminated buildings (*10*). A survey of 159 New Orleans area residents 7 weeks after Katrina found that 68% of those interviewed were aware of the recommendation (*11*) and that at least 30% of those participating in remediation activities had used a NIOSH-certified respirator (*12*). Despite these levels of awareness and experience, subsequent anecdotal reports suggested that some New Orleans residents were not properly donning N95 FF respirators. Improper donning would promote the entry of unfiltered air through leaks or gaps between the respirator and the skin, compromising the protection offered (*13*). To better understand respirator use by the public, we investigated the nonoccupational use and donning of N95 FF respirators in post-hurricane New Orleans.

## Methods

### Participants

Using geographic information system mapping software, we randomly selected homes in Orleans Parish (city of New Orleans). To focus on residential areas, we eliminated 6,345 of the parish’s 10,181 census blocks (US Census, 2000). The eliminated blocks were likely to be sparsely populated or to contain industrial buildings, commercial centers, or parks (blocks with <20 housing units and blocks in the lower 2.5% of housing unit density); to contain mostly high-rise apartment buildings or public housing units that would be difficult to access or remain uninhabited (blocks in the upper 2.5% of housing unit density); and to be in uninhabited neighborhoods (blocks in the Lower Ninth Ward). We randomly generated 600 waypoints (unique locations based on latitude and longitude) across the remaining 3,836 census blocks.

Each waypoint served as a starting point to locate eligible participants. Using a global positioning system device, a survey team navigated to a waypoint and identified the nearest home. To be eligible for participation, a person had to be an English-speaking adult (>18 years of age) associated with a home as its owner, current occupant, or relative/friend of the owner/occupant. Because we were interested in nonoccupational respirator use by the general public, persons at a home as paid employees (e.g., remediators) were not eligible. However, residents encountered at their own homes who were employed as remediators were not excluded. If unable to conduct an interview at the first encountered home, the team proceeded in a systematic fashion to the next home. Once 1 interview was conducted at a waypoint, the team navigated to the next waypoint and repeated the process.

### Questionnaire and Evaluation of Respirator Donning

From March 4 to March 11, 2006, survey team members interviewed participants with a 10-minute questionnaire that collected information on experience with residential flooding, water damage, and mold growth; participation in mold clean-up activities; lifetime and post-Katrina experiences with respiratory protection (both noncertified dust masks and surgical masks, hereafter “masks”; and NIOSH-certified respirators, including disposable and reusable types, hereafter “certified respirators”); and nonidentifying demographic factors, including self-identified ethnicity and race. Each team used the same photographs and actual examples of masks and certified respirators during the interviews.

**Figure 2:**
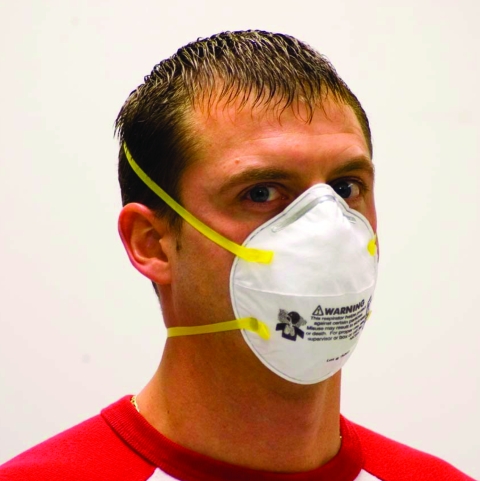
Properly donned disposable N95 filtering facepiece respirator. To be properly donned, the respirator must be correctly oriented on the face and held in position with both straps. The straps must be correctly placed, with the upper strap high on the head and the lower strap below the ears. For persons with long hair, the lower strap should be placed under (not over) the hair. The nose clip must be tightened to avoid gaps between the respirator and the skin. Facial hair should be removed before donning. Photo used with permission.

Each interview included an evaluation of respirator donning. Interviewers were trained before the survey on proper donning (Figure 2), including the following: proper orientation of the respirator; use of both straps; proper placement of straps; need for tightening of the nose clip; and need for removal of facial hair (*14*,*15*). A participant with an N95 FF respirator that appeared to be in good condition was permitted to use this respirator for the evaluation. Otherwise, the participant was asked to choose between 2 models then available from New Orleans retailers. The interviewer asked the participant to put on the N95 FF respirator as he or she would for participating in mold clean-up activities. Written and pictorial manufacturer’s instructions were included with the respirator packaging, but no additional instructions were given until the evaluation was complete (*16*). The interviewer recorded whether the participant referred to the manufacturer’s instructions and, once the participant indicated that the respirator was donned, noted any observed “donning errors” that could contribute to an insufficient fit.

### Statistical Analyses

For calculations of frequencies of donning errors, we included all participants who had at least 1 error. However, because 2 errors, visible gap and facial hair, could reflect aspects of study design (size and shape of respirators offered and lack of opportunity to shave before evaluation, respectively) rather than participants’ donning technique, they were not considered in analyses of factors associated with proper donning. For these analyses, participants with at least 1 of the other donning errors were categorized as improperly donning the respirator; the remaining participants were categorized as properly donning the respirator.

To identify factors associated with proper donning, we used contingency tables and simple logistic regression. We included significant factors (p<0.05) in multiple logistic regression models, applying stepwise logistic regression. We used the likelihood ratio χ^2^ test and calculated odds ratios (OR) with 95% likelihood confidence intervals (CI). We conducted analyses with SAS (version 9.1) and JMP (version 5.1) software packages (SAS Institute, Cary, NC, USA).

## Results

### Participants

We conducted 553 interviews at the 600 visited waypoints, for a response rate of 92%. Half of the participants were male, with a median age of 50 years, and about half identified their race as Caucasian and half as African-American or black (Table 1). Most described previously using a mask or respirator, but few reported ever having a respirator fit test. Most had participated in mold clean-up activities since Hurricane Katrina.

**Table 1:** Characteristics of Orleans Parish participants, March 2006*

Characteristic	Values
Age in y, median, range (N = 547)	50, 18–89
Male, n/N (%)	292/553 (53)
Hispanic, n/N (%)	21/548 (4)
Race, n/N (%)†	
Caucasian	241/548 (44)
African-American or black	296/548 (54)
Asian	20/548 (4)
American Indian or Alaska Native	21/548 (4)
Native Hawaiian or other Pacific Islander	5/548 (1)
Relationship to home, n/N (%)‡	
Owner	415/553 (75)
Renter	80/553 (14)
Other (includes relatives, friends, other associates)	58/553 (10)
Smoking status, n/N (%)	
Current	127/551 (23)
Former	123/551 (22)
Never	301/551 (55)
Physician-diagnosed asthma, n/N (%)	68/553 (12)
Flood level in feet,‡ median, range (N = 527)	4, 0–18
Water entry due to roof or window damage,‡ n/N (%)	300/547 (55)
Mold extent,‡ n/N (%)	
None	143/550 (26)
<50% of walls and ceilings	213/550 (39)
≥50% of walls and ceilings	179/550 (33)
Do not know	15/550 (3)
Employed in mold remediation, n/N (%)	45/553 (8)
Ever used mask or respirator, n/N (%)	439/553 (79)
Ever had respirator fit test,§ n/N (%)	80/543 (15)
Activities in water-damaged/moldy home since Katrina	
Been inside, n/N (%)	467/551 (85)
Participated in clean-up, n/N (%)	372/551 (68)
No. of homes cleaned (N = 368), median, range	2, 1–50
No. with mold extent ≥50% (N = 367), median, range	1, 0–25
Still participating in clean-up activities, n/N (%)	183/358 (51)

*Data for some characteristics were missing for some participants. †Participants could select >1 racial category; total >100%. ‡Home at which participant was encountered and interviewed. §“Fit test” was defined in the questionnaire as “a test in which a technician measures how well the respirator fits your face during activities such as talking and moving your head. It could involve smelling smoke, tasting something sweet or bitter, or a special machine that counts particles.”

### Respiratory Protection Use during Mold Clean-up Activities

Overall, of the 553 participants interviewed, 42% (n = 233) had used a certified respirator, and 35% (n = 192) had used an N95 FF respirator, specifically, for mold clean-up activities since Katrina. Among the 368 who reported participating in mold clean-up activities, most (n = 315, 86%) reported using a mask or certified respirator during those activities, most frequently the N95 FF respirator (Table 2). A minority (n = 60, 19%) of the 315 reported referring to the manufacturer’s instructions. More commonly (n = 129, 41%), participants stated that they used respiratory protection without any instruction.

**Table 2:** Orleans Parish participants’ experiences with respiratory protection during mold clean-up activities since Hurricane Katrina, March 2006*

Experience	n/N (%)
Used mask or respirator	315/368 (86)
Type of mask or respirator used†	
Noncertified mask (dust or surgical)	143/315 (45)
Certified respirator, type†	233/315 (74)
Disposable N95 filtering facepiece	192/233 (82)
Reusable half-face with cartridges	87/233 (37)
Reusable full-face with cartridges	4/233 (2)
Source of mask or respirator†	
Store	207/315 (66)
Nongovernmental organization‡	73/315 (23)
Workplace	27/315 (9)
Relative or friend	24/315 (8)
Other source	16/315 (5)
Main source of information on use of mask or respirator	
Manufacturer’s instructions	60/315 (19)
Media	20/315 (6)
Instructions given at work	51/315 (16)
Store employee/clerk	5/315 (2)
Relative or friend	16/315 (5)
Internet site§	10/315 (3)
Other source	24/315 (8)
No information used	129/315 (41)
Conditions that would prompt replacing mask or respirator†	
When it became dirty	163/312 (52)
When it became damaged	34/312 (11)
When it became harder to breathe through	25/312 (8)
Other¶	131/312 (42)

*372 (68%) of 553 survey participants reported participating in mold clean-up activities since Hurricane Katrina. Data for some characteristics were missing for some participants. †Participants could choose >1 response; total >100%. ‡Includes Red Cross, Salvation Army, volunteer groups, and church groups. §In 4 cases, Internet site was specified by name: Channel 6, Federal Emergency Management Agency, city of New Orleans, and National Institute for Occupational Safety and Health. ¶Write-in responses included various time intervals (e.g., every 3 h, daily, weekly, never) and other conditions such as when smelling moldy odor or feeling sick.

### Evaluation of Respirator Donning

A total of 538 (97%) participants agreed to put on an N95 FF respirator. Most of these (n = 489, 91%) used 1 of the 2 models offered by the interviewers. Twelve (2%) referred to the manufacturer’s directions.

Overall, 433 (80%) of the participants who donned an N95 FF respirator were noted to have at least 1 donning error that could contribute to a poor fit. More than half of these did not tighten the nose clip, and half incorrectly placed the 2 straps; in addition, 22% put the respirator on upside down, and 21% used only 1 strap (Table 3). While 31% (n = 135) made l error, 34% (n = 146) made 2, and 35% (n = 152) made >3.

**Table 3:** Errors observed among Orleans Parish participants donning disposable N95 filtering facepiece respirators, March 2006

Error	n (%)*
Nose clip not tightened	303 (71)
Both straps used, but straps incorrectly placed	221 (52)
Visible gap between respirator and skin†	136 (32)
Respirator donned upside down	94 (22)
Only 1 of 2 straps used	91 (21)
Facial hair†	48 (11)
Respirator donned sideways or tilted	11 (3)
Other‡	5 (1)

*N = 427; 433 participants were noted to have at least 1 donning error; for 427 participants, the nature of the error(s) was documented. †Among those participants with only 1 observed donning error, 6 had a visible gap between the respirator and skin, and 18 had facial hair. These 24 participants are included in calculations of frequencies of donning errors. For analyses of factors associated with proper donning, they were considered to have properly donned the respirator. ‡The “other” category was used for 2 participants who were noted to be unable to put on the respirator and for 1 participant who did not use either of the 2 straps. For 2 other participants, the “other” category was indicated, but the interviewer did not further specify the nature of the errors.

### Characteristics Associated with Proper Donning

For 24 participants, the only noted donning error was a visible gap (n = 6) or facial hair (n = 18). When these 24 persons who otherwise demonstrated proper donning were included, 129 (24%) of the participants properly donned the N95 FF respirator. In simple logistic regression analyses of all participants, proper donning was significantly associated with several personal factors: being male, being Caucasian, and being a nonrenter (i.e., a homeowner or associate) in the home at which the interview occurred. In addition, proper donning was associated with post-hurricane experiences: having been inside a water-damaged or moldy home and having participated in mold clean-up. Finally, proper donning was associated with several factors related to respirators: ever having used a mask or certified respirator, ever having had a respirator fit test, having at the time of the interview a mask or certified respirator, and having at the time of the interview a respirator confirmed by the interviewer to be NIOSH certified (Table 4).

**Table 4:** Characteristics associated with proper donning of disposable N95 filtering facepiece (FF) respirators in simple logistic regression analyses of all Orleans Parish participants and subsets of participants

Characteristic	Proper donning (%)*		OR (95% CI)†	p value
	With characteristic	Without characteristic		
All participants (N = 538)‡				
Male	34	12	3.84 (2.47–6.12)	<0.001
Caucasian (N = 533)	29	20	1.66 (1.12–2.49)	0.01
Relationship to interview home (nonrenter vs. renter)	26	13	2.29 (1.19–4.87)	0.01
Ever used mask or respirator	29	5	8.31 (3.57–23.44)	<0.001
Ever had respirator fit test	58	18	6.10 (3.69–10.21)	<0.001
Been inside water-damaged/moldy home (N = 536)	26	14	2.11 (1.12–4.35)	0.02
Participated in clean-up (N = 536)	29	14	2.58 (1.60–4.31)	<0.001
Mask or respirator at interview	38	18	2.74 (1.80–4.15)	<0.001
Confirmed certified respirator at interview	44	19	3.48 (2.23–5.43)	<0.001
Participated in clean-up§ (N = 363)¶				
Used mask or respirator during clean-up	31	16	2.42 (1.15–5.74)	0.02
Used certified respirator during clean-up	39	12	4.54 (2.59–8.42)	<0.001
Used N95 FF respirator during clean-up	35	22	2.02 (1.27–3.25)	<0.01
Used a mask or respirator during clean-up# (N = 312)**				
Workplace source of mask or respirator	48	29	2.22 (1.00–4.95)	0.05
Workplace source of information	47	28	2.29 (1.24–4.23)	<0.01

*Proportion of participants with characteristic demonstrating proper donning, followed by proportion of participants without characteristic demonstrating proper donning. For the first characteristic, 34% of males and 12% of females properly donned the respirator. †Unadjusted odds ratio (OR) and 95% confidence interval (CI) for proper donning of disposable N95 FF respirator by participants with characteristic compared to participants without characteristic. ‡538 of 553 participants donned an N95 FF respirator for the interviewer. For some analyses, N is <538 (as noted) because of missing data. §The following variables were also statistically significant in simple logistic regression analyses for this subset: male, Caucasian, ever used mask or respirator, ever had respirator fit test, had mask or respirator at time of interview, and had certified respirator at time of interview. ¶367 of 372 who participated in clean-up donned an N95 FF respirator for the interviewer. N is <367 because of missing data. #The following variables were also statistically significant in simple logistic regression analyses for this subset: male, Caucasian, ever had respirator fit test, had mask or respirator at time of interview, had certified respirator at time of interview, used certified respirator during clean-up, and used disposable N95 FF respirator during clean-up. **312 of 315 who used a mask or respirator during clean-up donned an N95 FF respirator for the interviewer.

For the subset that had participated in clean-up activities, proper donning was also associated with use of respiratory protection during clean-up, including having specifically used an N95 FF respirator. For the subset that had used a mask or certified respirator during clean-up, having obtained that mask or respirator from the workplace and having obtained information on how to use the mask or respirator from the workplace were also significant factors. Proper donning was not associated with age, Hispanic ethnicity, level of floodwater, water incursion due to roof or window damage, extent of mold coverage, current employment in mold remediation, asthma diagnosis, smoking status, or respirator brand.

When multiple logistic regression was used, the factors significantly associated with proper donning for all participants were as follows: ever having used a mask or certified respirator (OR 5.28; 95% CI, 1.79–22.64), ever having had a respirator fit test (OR 4.40; 95% CI, 2.52–7.81), being male (OR 2.44; 95% CI, 1.50–4.03), being Caucasian (OR 2.09; 95% CI, 1.32–3.33), having a certified respirator at the time of the interview (OR 1.99, 95% CI 1.20–3.28), and having participated in mold clean-up activities (OR 1.82; 95% CI,1.00–3.41). For the subset that participated in mold clean-up, the significant factors were as follows: having used a certified respirator during clean-up (OR 5.17; 95% CI, 2.75–10.24); ever having had a respirator fit test (OR 3.38; 95% CI, 1.75–6.61); being Caucasian (OR 3.38, 95% CI, 1.97–5.91); and being male (OR 2.80; 95% CI, 1.58–5.13). These same factors were also significant for the subset that used a mask or certified respirator during clean-up.

## Discussion

The protection afforded by a certified respirator depends on its fit, and a fundamental component of achieving a good fit is proper donning (*13*,*17*). In post-hurricane New Orleans, public concern about adverse health effects of exposure to mold was near universal (*11*,*12*). Yet our investigation demonstrated that, despite this high level of motivation, most of participants did not properly don an N95 FF respirator.

Our investigation benefited from several strengths. We used a random selection process to obtain our sample, and comparisons with existing population-based surveys suggest we achieved a representative cross-section (*18*,*19*). Given inconsistencies in respiratory protection terminology, we facilitated effective communication by using photographs and actual examples of masks and certified respirators. Finally, of the few prior field investigations that have addressed N95 FF respirator donning (*20*–*22*), none have focused on nonoccupational use.

An important limitation is that we did not confirm the observed proper donnings with respirator fit testing. While we cannot estimate the relative contribution of each donning error to declining protection without quantitative measurements, those participants who put on the respirator improperly would clearly have failed a standard fit test. However, the outcome in those who appeared to properly put on the respirator is less certain because even a properly donned respirator may have leaks that limit its effectiveness. A study of 18 different N95 FF respirator models found that, overall, in the absence of fit testing, 74% of proper donnings would provide the full protection possible with an N95 FF respirator (range 31%–99%, depending on the model) (*23*). Thus, the proportion of our participants who would have achieved the full protection possible with an N95 FF respirator is likely to be lower than the proportion who demonstrated proper donning. Ultimately, designing models with good fit characteristics would be beneficial.

Our findings have implications for the use of N95 FF respirators by members of the public to prevent the transmission of communicable diseases. Both experimental and epidemiologic studies suggest that airborne transmission of influenza (by small particles <10 μm) can occur and may result in more severe disease than transmission by large droplets or fomites (*24*–*27*). A recent review argues that airborne transmission may play an important role in the spread of a pandemic strain (*28*). While formal recommendations for N95 FF respirator use by the public do not exist—beyond the HHS recommendation regarding potential exposures to infected birds—a recent Institute of Medicine (IOM) report notes that “a properly fitted N95 FF respirator is likely to be both the least expensive and the most widely available NIOSH-certified respirator for protecting... the public against airborne [influenza] infection” (*29*). Our results suggest that much of the public may have difficulty achieving a proper fit because of improper donning. Given the observed role of experience in proper donning, and the high frequency of recent experience with respirators reported by our survey participants, one could argue that the overall performance in post-Katrina New Orleans is likely to be superior to that of virtually any other locale.

The World Health Organization anticipates use of respiratory protection by the public will occur spontaneously in the event of pandemic influenza (*30*,*31*). Indeed, N95 FF respirators are currently being marketed to the public as “bird flu masks” (*32*). While uncertainty remains about the level of protection needed against influenza and that offered by an N95 FF respirator, an improperly donned N95 FF respirator will provide less protection than a properly donned one. Our results suggesting that workplace training increased proper donning among the public indicate that educational efforts could have a positive effect. Since few of our participants reported, or were observed, referring to manufacturers’ instructions, consideration also should be given to incorporating instructions onto the respirator itself, such as arrows or simple words (“nose,” “chin”) to indicate orientation. The IOM report’s authors could find no simple modification of N95 FF respirators that would prevent the need for fit testing (*29*). Short of mass fit testing, proper donning will be the vital step to ensuring that members of the public using N95 FF respirators derive the greatest possible benefit from them.

Even under workplace conditions, respirator donning may be imperfect. An observational study of 62 healthcare workers in 3 California hospitals found that 40 (65%) improperly put on N95 FF respirators before entering the room of a patient in isolation for tuberculosis. Errors included use of only 1 strap, incorrectly placed straps, and presence of facial hair (*21*). The results of that study, in terms of the proportion who demonstrated improper donning and the nature of the errors, are similar to our findings. The impact of the 2005 US policy that suspended enforcement of annual fit testing of healthcare workers who use respirators for occupational exposure to tuberculosis is unknown (*33*). Yet N95 FF respirators will clearly be part of healthcare workers’ defense in the event of pandemic influenza (*34*,*35*). HHS, as part of procurement of essential medical supplies for pandemic influenza, has stockpiled 20 million N95 FF respirators and plans to acquire 87 million more through September 2007 (*36*). The pandemic plans of other countries, including Australia and France, recommend use of N95 (or FFP2) respirators (*28*). Further evaluation of respirator donning among healthcare workers therefore may be warranted.

In summary, this population-based survey of nonoccupational respirator use found that a minority of participants demonstrated proper donning of an N95 FF respirator. Our findings are of particular importance to public health agencies planning for future events, from floods to pandemic influenza, in which use of N95 FF respirators by the public will be recommended or is anticipated. A unique opportunity exists to enhance protection of the public through interventions, such as educational campaigns, training sessions, and respirator design modifications, aimed at improving the public’s ability to don a respirator correctly. Infection control officers and the healthcare workers they protect also may benefit from the insights gained from this survey.
